# Novel insights into the association between seasonal variations, blood pressure, and blood pressure variability in patients with new-onset essential hypertension

**DOI:** 10.1186/s12872-022-02840-1

**Published:** 2022-09-08

**Authors:** Long Tang, Jingshui Zhang, Yanan Xu, Tingting Xu, Yi Yang, Jun Wang

**Affiliations:** 1 Department of Cardiology, The People’s Hospital of Xuancheng City, Anhui, 242000 China; 2 Respiratory medicine department, The People’s Hospital of Xuancheng City, Anhui, 242000 China; 3 Dermatology department, The People’s Hospital of Xuancheng City, Anhui, 242000 China; 4 Department of Cardiology Fourth Ward, the Xinjiang Medical University Affiliated Hospital of Traditional Chinese Medicine, Ürümqi, 830011 China

**Keywords:** Seasonal variations, Essential hypertension, Blood pressure variability, Heart rate variability

## Abstract

**Background:**

Blood pressure (BP) exhibits seasonal variations, with peaks reported in winter. However, the association between seasonal variations and blood pressure variability in patients with new-onset essential hypertension is not fully understood. This study evaluated the potential association of seasonal variations with new-onset essential hypertension.

**Methods:**

This retrospective observational study recruited a total of 440 consecutive patients with new-onset essential hypertension who underwent 24-h ambulatory electrocardiograph (ECG) and BP measurement at our department between January 2019 and December 2019. Demographic and baseline clinical data including BP variability, heart rate variability, and blood tests were retrieved. Multivariate linear regression analysis was performed to identify factors independently associated with mean BP and BP variability.

**Results:**

Among the 440 patients recruited, 93 cases were admitted in spring, 72 in summer, 151 in autumn, and 124 in winter. Univariate analysis revealed that systolic BP (SBP), diastolic BP (DBP), high-sensitivity C-reactive protein, SBP drop rate, DBP drop rate, 24-h standard deviation of SBP, 24-h standard deviation of DBP, 24-h SBP coefficient of variation, and 24-h DBP coefficient of variation were associated with patients admitted in winter (*P* < 0.05 for all). Multivariate linear regression analysis showed that winter was the influencing factor of 24-h standard deviation of SBP (*B* = 1.851, *t* = 3.719, *P* < 0.001), 24-h standard deviation of DBP (*B* = 1.176, *t* = 2.917, *P* = 0.004), 24-h SBP coefficient of variation (*B* = 0.015, *t* = 3.670, *P* < 0.001), and 24-h DBP coefficient of variation (*B* = 0.016, *t* = 2.849, *P* = 0.005) in hypertensive patients.

**Conclusions:**

Seasonal variations are closely associated with BP variability in patients with new-onset essential hypertension. Our study provides insight into the underlying pathogenesis of new-onset essential hypertension.

## Introduction

Essential hypertension, also known as primary hypertension, is the most common risk factor for cardiovascular diseases [1, 2]. Therefore, it is imperative to control hypertension in daily life. It has been well documented that many factors, such as age, smoking, and dietary habits, can affect new-onset essential hypertension. Seasonal variations have also been proposed as an additional risk factor. For example, higher environmental temperatures have been linked to lower blood pressure (BP), while lower temperatures have been linked to higher BP [3]. Although an association between seasonal variations and BP fluctuations has long been noted, there is very little agreement regarding how to manage the influence of seasonal changes on BP [3–9]. It should be pointed out that the effects of seasonal variations could be easily overlooked, leading to failed BP control and often catastrophic adverse events in clinical practice [4].

Previous studies have shown that cardiovascular risk can be appropriately managed by monitoring BP and seasonal changes [4, 8]. Of note, there is evidence supporting an association between BP fluctuation and environmental temperatures [3]. Current clinical and experimental data indicate that there are correlations between winter and autonomic nervous system activation and that a rise in peripheral vascular resistance contributes to the onset of essential hypertension [3]. In addition, BP variability has received much attention because of the wide application of 24-h ambulatory BP monitoring. A higher BP variability serves as a validated predictor of increased risk of cardiovascular events, independently of the average BP values [10]. However, to the best of our knowledge, the potential relationships between mean BP levels, BP variability, and seasonal variations in patients with new-onset essential hypertension have not been well examined. In the present study, we sought to systematically evaluate associations between seasonal variations and aspects of new-onset essential hypertension.

## Methods

### Patient recruitment and study design

**Fig. 1:**
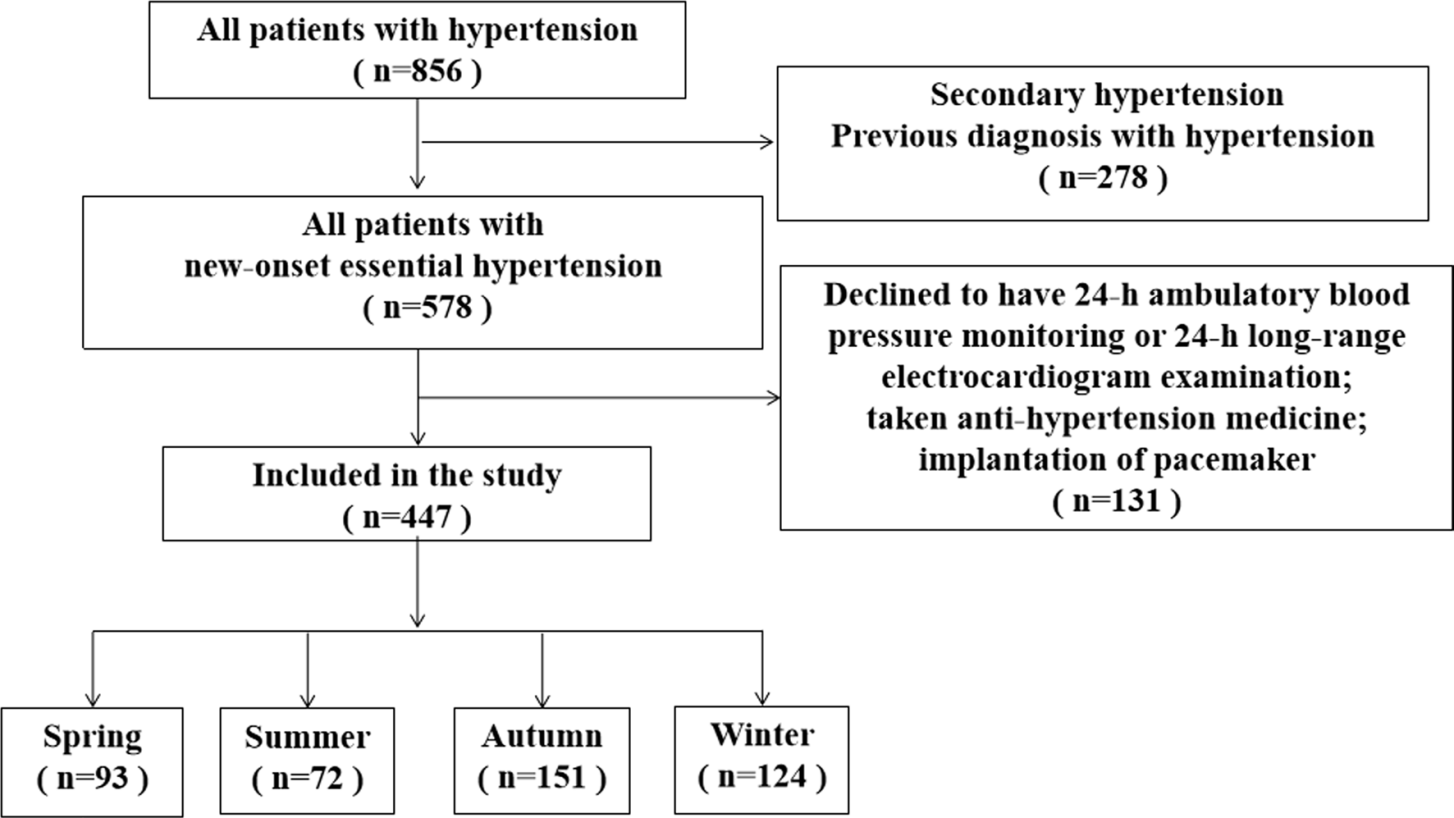
Flowchart of patient enrollment

This retrospective observational study recruited 440 consecutive patients with a first diagnosis of essential hypertension who were scheduled to receive 24-h ambulatory electrocardiograph (ECG) and BP measurement at the People's Hospital of Xuancheng City between January 2019 and December 2019. These patients included 93 who were admitted in spring, 72 in summer, 151 in autumn, and 124 in winter. Diagnostic criteria of essential hypertension conformed to criteria recently described in clinical methodology: BP measured at home three times on different days with a systolic BP (SBP) > 140 mmHg or diastolic BP (DBP) > 90 mmHg [11]. Patients were excluded if they had any of the following clinical conditions: previous diagnosis of hypertension, secondary hypertension, declined to accept 24-h ambulatory ECG, BP measurement with non-effective readings > 20%, medications that affected BP or heart rate before admission, acute coronary syndrome, implantation of pacemaker, stroke, cardiopulmonary insufficiency, cancer, any autoimmune diseases, any hematological diseases, severe infection, or acute mental or psychological stress. The study was approved by the Ethics Committee of The People's Hospital of Xuancheng City (No. 2022-1w002-01) before the performance. Because this was a retrospective observational study, the ethics committee of The People's Hospital of Xuancheng City waived the requirement for informed consent from eligible patients. All methods were performed in accordance with the relevant guidelines and regulations. Figure 1 shows the flow chart of patient selection in this study.

### 24-h ambulatory ECG and BP measurement

All patients underwent 24-h ambulatory ECG and BP monitoring (CB-2304-A/CB-2306-A, VasoMedical, China) upon admission to the Department of Cardiology. 24-h ambulatory BP monitoring was performed using a cuff-monitor. BP was measured every 25 min during the day (between 08:00 and 22:00) and every 60 min during the night (between 22:00 and 08:00 the next morning) using a cuff size that fit the individual’s arm circumference as previously described [12]. Only recordings rated as of sufficient quality, defined as at least 80% of valid readings over the 24-h period, were included for analysis; at least 20 valid daytime and at least 7 valid nighttime measurements were required. All participants were requested to maintain their normal diet and to refrain from vigorous exercise, alcohol, smoking, and caffeinated beverages the day of the test. The following parameters were recorded: 24-h ambulatory BP, containing 24-h average SBP, 24-h SBP load, 24-h DBP, 24-h DBP load, 24-h average BP, 24-h pulse pressure difference, 24-h SBP standard deviation, 24-h DBP standard deviation, 24-h SBP coefficient of variation, 24-h DBP coefficient of variation, ambulatory arterial stiffness index (AASI), heart rate variability, containing standard deviation of normal R-R intervals (SDNN), standard deviation average of NN intervals (SDANN), root mean square successive difference of normal R-R intervals (rMSSD), percent of the number of times that the difference between adjacent normal RR intervals > 50 ms in the total number of NN intervals (PNN50), high-frequency power (HF), low-frequency power (LF), and LF/HF ratio. All parameters were calculated according to previously published methods using customized and validated software [13, 14]. 24-h SBP coefficient of variation = (24-h SBP standard deviation) / (24-h average SBP; 24-h DBP coefficient of variation = (24-h DBP standard deviation) / (24-h average DBP). BP load was defined as the percentage of readings greater than the normal in total measurements, based on a daytime SBP > 140 mmHg or DBP > 90 mmHg and a nighttime SBP > 120 mmHg or DBP > 80 mmHg. The SBP drop rate = [(mean daytime SBP – mean night SBP) / mean daytime SBP]*100, and the DBP drop rate = [(mean daytime DBP—mean night DBP) / mean daytime DBP]*100. The SDNN and SDANN were regarded as vagal and sympathetic influences [14]. rMSSD and pNN50 were regarded as parasympathetic nerve activity [15]. For frequency domain variables, HF generally reflects cardiac parasympathetic nerve activity, while LF is possibly correlated with sympathetic tone [15]. The LF/HF ratio is an indicator of the balance between sympathetic and parasympathetic activities, with higher LF/HF ratios indicating increased sympathetic nerve excitability [15].

#### Definition

The date of 24-h ambulatory BP monitoring was used to define the seasons as follows: spring: March, April, May; summer: June, July, August; autumn: September, October, November; and winter: December, January, February [16]. Data on meteorological parameters such as average temperatures, the maximum and minimum temperatures, and precipitation during each BP monitoring period were obtained from the Meteorology Bureau of Xuancheng (http://ah.cma.gov.cn/). Smoking was defined as current smoker. Educational qualifications were classified as illiterate, primary school, middle school, high school, specific college course, or above.

### Statistical analysis

Normally distributed continuous variables are presented as mean ± standard deviation (SD), and medians and interquartile ranges (IQRs) are otherwise presented. Categorical variables are presented as percentages. A chi-square (χ^2^) test was used to analyze the differences in categorical variables between groups, and analysis of variance (ANOVA) was used for comparisons of data among multiple groups. The Mann–Whitney U test or Kruskal–Wallis variance analysis was used for non-normally distributed data between groups. The multivariate linear regression analysis was conducted with 24-h average SBP, 24-h average DBP, 24-h standard deviation of SBP, 24-h standard deviation of DBP, 24-h SBP coefficient of variation, or 24-h DBP coefficient of variation as the dependent variable, and the variables that were statistically significant in the univariate analysis were considered independent variables. Multiple linear regression analysis was used to study the association of seasonal variations with mean BP levels and BP coefficient of variation. A P-value less than 0.05 was considered statistically significant. All statistical analyses were performed using SPSS23 ( IBM).

## Results

### Demographic and baseline clinical characteristics of patients

A total of 440 patients were registered in this study. The patients were divided into four groups according to the dates of ECG and BP measurements: 93 in spring, 72 in summer, 151 in autumn, and 124 in winter. The demographic and baseline clinical characteristics and biochemical parameters of patients in these four groups are presented in Table 1. There were no differences in the demographic and baseline clinical characteristics between these four groups. However, compared with other groups, the winter group were more likely to have higher clinic SBP (*P* < 0.001), higher clinic DBP (*P* < 0.001), higher high-sensitivity C-reactive protein (hs-CRP) (*P* = 0.003), lower maximum temperature (*P* < 0.001), lower minimum temperature (*P* < 0.001), lower average temperature (*P* < 0.001), and lower mean precipitation.

**Table 1:** Demographic and baseline clinical characteristics of patients

	Spring (*n* = 93)	Summer (*n* = 72)	Autumn (*n* = 151)	Winter (*n* = 124)	*t*/Z/*χ*^2^	*P*
Male n (%)	53 (57.0)	45 (62.5)	87 (57.6)	75 (60.5)	0.752	0.861
Age (years)	60.4 ± 13.3	57.7 ± 12.9	59.6 ± 12.9	56.3 ± 11.3	2.427	0.065
Diabetes mellitus	13 (14.0)	14 (19.4)	16 (10.6)	14 (11.3)	3.825	0.281
Duration of diabetes mellitus (years)	10.00 (7.53,10.00)	3.50 (0.83,6.25)	2.00 (2.00,9.25)	8.00 (3.25,12.00)	3.756	0.289
Current smoker n (%)	22 (23.7)	16 (22.2)	30 (19.9)	21 (16.9)	1.700	0.637
Duration of smoker (years)	22 (23.7)	16 (22.2)	30 (19.9)	21 (16.9)	1.700	0.637
CAD n (%)	3 (3.2)	5 (6.9)	6 (4.0)	3 (2.4)	2.411	0.492
PCI n (%)	0 (0.0)	2 (2.8)	4 (2.6)	4 (3.2)	4.894	0.180
Previous myocardial infarction *n* (%)	1 (1.1)	3 (4.2)	5 (3.3)	1 (0.8)	3.915	0.271
Family history of hypertension *n* (%)	3 (3.2)	5 (6.9)	6 (4.0)	3 (2.4)	2.411	0.492
Educational Qualifications *n* (%)					20.007	0.067
Illiterate	18 (19.4)	13 (18.1)	34 (22.5)	19 (15.3)		
Primary school	22 (23.7)	25 (34.7)	27 (17.9)	31 (25.0)		
Middle school	36 (38.7)	17 (23.6)	47 (31.1)	36 (29.0)		
High school	6 (6.5)	6 (8.3)	18 (11.9)	23 (18.5)		
Specific colleague course or above	11 (11.8)	11 (15.3)	25 (16.6)	15 (12.1)		
BMI (kg/m^2^)	26.06 ± 5.67	25.53 ± 3.99	25.47 ± 3.96	25.55 ± 3.81	0.390	0.760
Clinical SBP (mmHg)	156.3 ± 24.2	150.9 ± 24.9	156.9 ± 21.4	164.3 ± 22.2	5.738	0.001
Clinical DBP (mmHg)	93.8 ± 14.9	89.4 ± 12.4	95.4 ± 14.0	100.1 ± 15.1	9.100	< 0.001
hs-CRP (mg/L)	0.61 (0.50,0.99)	0.53 (0.50,1.02)	0.60 (0.50,0.89)	0.79 (0.56,1.09)	13.651	0.003
HDL-C (mmol/L)	1.14 ± 0.33	1.21 ± 0.34	1.19 ± 0.27	1.15 ± 0.25	1.063	0.364
LDL-C (mmol/L)	2.78 ± 0.89	2.77 ± 0.85	2.65 ± 0.69	2.77 ± 0.79	0.792	0.499
TC (mmol/L)	4.49 ± 1.13	4.64 ± 0.93	4.58 ± 0.93	4.76 ± 1.08	1.363	0.253
TG (mmol/L)	1.53 (1.03,2.24)	1.37 (0.98,2.22)	1.44 (0.08,1.90)	1.28 (1.00,1.88)	3.468	0.325
ApoA1 (g/L)	1.26 ± 0.21	1.25 ± 0.22	1.27 ± 0.33	1.32 ± 0.25	1.524	0.208
ApoB (g/L)	1.08 ± 0.38	1.04 ± 0.25	1.07 ± 0.29	1.11 ± 0.31	0.652	0.582
Lp (a) (g/L)	14.60 (9.85,27.95)	16.05 (9.08,31.83)	13.00 (7.30,26.30)	16.05 (8.65,30.70)	2.297	0.513
Creatinine (mmol/L)	74.44 ± 28.37	71.75 ± 17.51	70.08 ± 17.81	68.00 ± 20.68	1.740	0.158
Uric acid (mmol/L)	355.29 ± 86.97	334.89 ± 82.80	338.52 ± 89.76	343.52 ± 84.53	0.971	0.406
Fasting glucose (mmol/L)	5.49 ± 0.92	5.66 ± 1.10	5.72 ± 1.29	5.73 ± 1.40	0.846	0.469
*Temperature*						
Maximum, °C	19.5 ± 4.7	30.9 ± 3.0	23.2 ± 3.9	10.2 ± 2.3	559.118	< 0.001
Minimum, °C	9.6 ± 4.3	23.1 ± 2.9	14.0 ± 4.4	3.0 ± 1.0	546.774	< 0.001
Average temperature °C	14.0 ± 4.5	26.5 ± 2.9	18.0 ± 4.2	6.1 ± 1.4	560.699	< 0.001
Mean precipitation (mm)	160.7 (70.1,60.7)	146.7 (43.7,526.9)	65.8 (10.5,66.3)	88.4 (23.6,150.3)	120.572	< 0.001

*BMI* Body mass index, *SBP* Systolic blood pressure, *DBP* Diastolic blood pressure, *TC* Total cholesterol, *TG* Triglyceride, *HDL-c* High-density lipoprotein cholesterol, *LDL-c* Low-density lipoprotein-cholesterol, *Apo-AI* Apolipoprotein A1, *Apo-B* Apolipoprotein B, *Lp(a)* Lipoprotein (a), *CAD* Coronary artery disease, *hs-CRP* High-sensitivity C-reactive protein, *PCI* Percutaneous transluminal coronary intervention

### 24-h ambulatory ECG and BP measurements of patients

The winter group had the highest SBP drop rate, DBP drop rate, 24-h SBP standard deviation, 24-h DBP standard deviation, 24-h SBP coefficient of variation, and 24-h SBP coefficient of variation among the four groups (all *P* < 0.05; Table 2). 24-h SBP standard deviation, 24-h DBP standard deviation, 24-h SBP coefficient of variation, and 24-h SBP coefficient of variation were higher in winter than in spring, summer, and autumn (all *P* < 0.05). Heart rate, mean daytime heart rate, LF, and HF were higher in summer and winter than in spring and autumn (all *P* < 0.05).

**Table 2:** 24-h ambulatory ECG and BP

	Spring (*n* = 93)	Summer (*n* = 72)	Autumn (*n* = 151)	Winter (*n* = 124)	*t*/Z/*χ*^2^	*P*
Mean daytime SBP (mmHg)	136.09 ± 14.67	136.04 ± 12.56	135.22 ± 12.86	138.09 ± 12.29	1.136	0.334
Mean daytime DBP (mmHg)	79.09 ± 11.44	79.43 ± 8.73	79.69 ± 9.77	82.55 ± 12.50	2.494	0.059
Mean daytime BP (mmHg)	100.09 ± 11.67	102.19 ± 10.72	100.93 ± 10.01	101.45 ± 10.97	0.580	0.628
Mean night SBP (mmHg)	129.47 ± 14.55	128.75 ± 15.46	129.58 ± 14.83	127.78 ± 15.70	0.375	0.711
Mean night DBP (mmHg)	73.94 ± 10.57	74.38 ± 10.72	75.21 ± 11.09	74.71 ± 10.79	0.287	0.835
Mean night BP (mmHg)	96.10 ± 13.14	95.31 ± 12.22	96.53 ± 14.35	96.20 ± 12.45	0.140	0.936
All-day average SBP	134.18 ± 15.90	135.42 ± 13.99	133.81 ± 13.83	134.68 ± 14.12	0.227	0.877
All-day SBP load	54.76 (29.73,76.19)	61.94 (38.16,80.00)	55.26 (34.48,79.41)	48.29 (21.89,71.79)	7.199	0.066
All-day average DBP	77.95 ± 10.49	80.17 ± 12.99	78.38 ± 10.42	79.65 ± 11.04	0.843	0.471
All-day DBP load	31.71 (6.65,53.58)	40.93 (22.92,67.68)	44.40 (21.21,64.71)	29.98 (14.63,54.04)	7.019	0.071
All-day average BP	99.23 ± 11.25	99.35 ± 11.67	99.77 ± 10.60	99.49 ± 13.42	0.047	0.986
All-day pulse pressure difference	56.77 ± 9.25	56.68 ± 10.35	55.52 ± 10.25	55.36 ± 10.22	0.498	0.684
24 h SBP standard deviation	14.69 ± 3.48	14.03 ± 3.78	14.38 ± 3.36	15.81 ± 3.06	5.781	0.001
24 h DBP standard deviation	11.68 ± 3.03	11.36 ± 2.45	11.39 ± 2.80	12.50 ± 2.67	4.388	0.005
24 h SBP coefficient of variation	0.11 ± 0.03	0.10 ± 0.03	0.11 ± 0.03	0.12 ± 0.03	4.998	0.002
24 h DBP coefficient of variation	0.15 ± 0.04	0.14 ± 0.03	0.15 ± 0.04	0.16 ± 0.04	3.072	0.028
Ambulatory arterial stiffness index	0.36 (0.24,0.48)	0.44 (0.26,0.55)	0.39 (0.23,0.53)	0.38 (0.26,0.54)	3.220	0.359
SDNN	129.31 ± 37.47	127.17 ± 34.16	130.27 ± 34.38	124.02 ± 32.32	0.822	0.482
SDANN	120.98 ± 58.91	111.29 ± 28.93	114.44 ± 31.78	110.45 ± 30.64	1.464	0.224
rMSSD	32.00 (21.94,47.88)	32.27 (22.03,50.51)	31.37 (24.54,45.19)	35.91 (25.79,52.11)	3.991	0.262
Pnn50	5.00 (1.75,10.93)	3.79 (2.11,8.90)	4.94 (2.07,11.31)	4.24 (2.01,10.02)	0.721	0.868
LF	2277.66 (1106.54,6613.00)	4556.99 (2319.30,9354.52)	2478.48 (1269.08,4790.46)	4458.33 (2659.93,9986.65)	33.653	< 0.001
HF	1103.22 (420.44,3464.37)	2196.51 (1011.66,5451.20)	1079.45 (481.41,3041.69)	2458.46 (972.65,5476.29)	27.529	< 0.001
LF/HF	2.06 (1.33,2.81)	2.07 (1.40,2.73)	2.09 (1.42,2.98)	1.98 (1.59,2.72)	0.895	0.827
Average heart rate (beats/min)	72.46 ± 10.91	75.79 ± 8.97	71.53 ± 8.93	75.38 ± 10.32	5.228	0.001
Mean daytime heart rate (beats/min)	77.04 ± 12.68	81.73 ± 11.41	76.61 ± 9.89	80.36 ± 11.28	4.775	0.003
Average night heart rate (beats/min)	66.03 ± 10.48	68.36 ± 8.42	66.17 ± 9.23	67.45 ± 9.75	1.130	0.337

### Linear regression analysis of 24-h average SBP

The demographic and baseline clinic characteristics and laboratory data of patients based on 24-h average SBP are shown in Table 3. Those with higher 24-h average SBP were mostly male, current smoker, and higher educational participants (all *P* < 0.05; (Table 4), Table 5). Multivariate linear regression analysis revealed that educational qualifications were independently and positively associated with 24-h average SBP (*P* < 0.05; Table 3).

**Table 3:** Univariate and multivariate linear regression models for 24-h average SBP

	Univariate			Multivariate		
	*β*	*t*	*P*	*β*	*t*	*P*
Female *n* (%)	− 2.738	1.973	0.049	− 0.979	0.633	0.527
Age (years)	− 0.015	0.285	0.776			
Diabetes mellitus	− 0.293	0.144	0.886			
Current smoker *n* (%)	3.730	2.198	0.028	3.023	1.675	0.095
CAD *n* (%)	− 4.401	1.954	0.051			
PCI *n* (%)	1.847	0.402	0.688			
Previous myocardial infarction *n* (%)	− 4.191	0.021	0.983			
Family history of hypertension *n* (%)	5.769	1.627	0.104			
Educational Qualifications n (%)	1.304	2.452	0.009	1.110	1.990	0.047
BMI (kg/m2)	0.218	0.905	0.367			
hs-CRP (mg/L)	− 0.264	0.231	0.763			
HDL-C (mmol/L)	− 1.923	0.809	0.419			
LDL-C (mmol/L)	0.733	0.843	0.399			
TC (mmol/L)	0.089	0.132	0.895			
TG (mmol/L)	0.638	1.056	0.292			
ApoA1 (g/L)	− 3.935	1.546	0.123			
ApoB (g/L)	1.809	0.818	0.414			
Lp (a) (g/L)	0.009	0.410	0.682			
Creatinine (mmol/L)	0.078	1.428	0.163			
Uric acid (mmol/L)	0.014	1.764	0.078			
Fasting glucose (mmol/L)	0.059	1.230	0.219			
*Season classification*						
Spring VS Summer	− 1.234	0.546	0.585			
Autumn VS Summer	− 1.609	0.780	0.436			
Winter VS Summer	− 0.739	0.347	0.729

**Table 4:** Univariate and multivariate linear regression model for 24-h average DBP

	Univariate			Multivariate		
	*β*	*t*	*P*	*β*	*t*	*P*
Female *n* (%)	− 2.393	2.242	0.025	− 0.014	0.284	0.777
Age (years)	− 0.339	8.790	< 0.001	− 0.354	7.200	< 0.001
Diabetes mellitus	1.459	0.929	0.353			
Current smoker *n* (%)	4.362	3.362	0.001	0.096	2.040	0.042
CAD n (%)	− 3.865	2.232	0.026	− 0.069	1.573	0.116
PCI n (%)	− 3.619	1.023	0.307			
Previous myocardial infarction n (%)	− 3.414	0.965	0.335			
Family history of hypertension n (%)	0.061	1.278	0.202			
Educational Qualifications n (%)	1.890	4.699	< 0.001	0.070	1.408	0.160
BMI (kg/m^2^)	0.342	2.836	0.005			
hs-CRP (mg/L)	− 0.466	0.531	0.596			
HDL-C (mmol/L)	− 2.072	1.133	0.258			
LDL-C (mmol/L)	0.610	0.912	0.363			
TC (mmol/L)	0.527	1.016	0.310			
TG (mmol/L)	1.112	2.400	0.017	0.009	0.208	0.835
ApoA1 (g/L)	-2.569	1.309	0.191			
ApoB (g/L)	2.640	1.554	0.121			
Lp (a) (g/L)	− 0.007	0.445	0.657			
Creatinine (mmol/L)	0.028	1.109	0.268			
Uric acid (mmol/L)	− 0.002	0.309	0.758			
Fasting glucose (mmol/L)	0.131	0.302	0.763			
*Season classification*						
Spring VS Summer	− 2.220	− 1.278	0.202			
Autumn VS Summer	− 1.789	− 1.129	0.259			
Winter VS Summer	− 0.522	− 0.318	0.751

**Table 5:** Univariate and multivariate linear regression models for 24-h standard deviation of SBP

	Univariate			Multivariate		
	*β*	*t*	*P*	*β*	*t*	*P*
Female *n* (%)	− 0.211	0.633	0.527			
Age (years)	0.028	2.154	0.032	0.025	1.783	0.075
Diabetes mellitus	− 0.207	0.424	0.672			
Current smoker *n* (%)	− 0.029	0.071	0.944			
CAD *n* (%)	0.365	0.676	0.499			
PCI *n* (%)	− 0.427	0.389	0.698			
Previous myocardial infarction *n* (%)	− 2.004	1.829	0.068			
Family history of hypertension *n* (%)	− 0.759	− 0.894	0.372			
Educational Qualifications *n* (%)	− 0.262	2.056	0.040	− 0.181	1.312	0.190
BMI (kg/m2)	-0.052	1.050	0.295			
hs-CRP (mg/L)	0.172	0.629	0.530			
HDL-C (mmol/L)	0.825	1.454	0.147			
LDL-C (mmol/L)	0.145	0.697	0.486			
TC (mmol/L)	0.054	0.333	0.740			
TG (mmol/L)	− 0.247	1.714	0.087			
ApoA1 (g/L)	0.885	1.453	0.147			
ApoB (g/L)	− 0.226	0.428	0.669			
Lp (a) (g/L)	0.001	0.129	0.897			
Creatinine (mmol/L)	0.001	0.125	0.901			
Uric acid (mmol/L)	− 0.001	0.541	0.589			
Fasting glucose (mmol/L)	0.018	0.134	0.894			
*Season classification*						
Spring VS Summer	0.661	1.246	0.213	0.592	1.121	0.263
Autumn VS Summer	0.347	0.717	0.474	0.323	0.671	0.503
Winter VS Summer	1.783	3.561	< 0.001	1.851	3.719	< 0.001

### Linear regression analysis of 24-h average DBP

The demographic and clinic characteristics and laboratory data of patients based on 24-h average DBP are shown in Table 4. Those with higher 24-h average DBP were mostly male and current smoker and had lower age and higher triglyceride levels without coronary artery disease (all *P* < 0.05; Table 4). Multivariate linear regression analysis revealed that age and smoking were independently associated with 24-h average DBP (all *P* < 0.05; Table 4).

### Linear regression analysis of 24-h standard deviation of SBP

The demographic and clinic characteristics and laboratory data of patients based on 24-h standard deviation of SBP are presented in Table 5. Those with higher 24-h average SBP were mostly older, had lower educational qualifications, and were admitted in winter (all *P* < 0.05; Table 5). Multivariate linear regression analysis showed that winter was independently associated with 24-h standard deviation of SBP (P < 0.05; Table 5).

### Linear regression analysis of 24-h standard deviation of DBP

The demographic and clinical characteristics and laboratory data of patients based on 24-h standard deviation of DBP are shown in Table 6. Those with 24-h standard deviation of DBP were mostly current smoker and had higher body mass index (BMI) and admitted in winter (all *P* < 0.05; Table 6). Multivariate linear regression analysis showed that current smoker, BMI, and winter were independently associated with 24-h standard deviation of DBP (*P* < 0.05; Table 6).

**Table 6:** Univariate and multivariate linear regression models for 24-h standard deviation of DBP

	Univariate			Multivariate		
	*β*	*t*	*P*	*β*	*t*	*P*
Female *n* (%)	− 0.525	1.947	0.052			
Age (years)	− 0.013	1.211	0.227			
Diabetes mellitus	0.372	0.939	0.348			
Current smoker *n* (%)	0.850	2.580	0.010	0.843	2.603	0.010
CAD *n* (%)	0.199	0.453	0.651			
PCI *n* (%)	0.056	0.063	0.950			
Previous myocardial infarction *n* (%)	− 0.582	0.651	0.516			
Family history of hypertension *n* (%)	− 1.219	1.769	0.078			
Educational Qualifications n (%)	− 0.011	0.106	0.915			
BMI (kg/m2)	0.091	2.996	0.003	0.086	2.871	0.004
hs-CRP (mg/L)	0.267	1.203	0.230			
HDL-C (mmol/L)	0.451	0.976	0.330			
LDL-C (mmol/L)	0.081	0.480	0.632			
TC (mmol/L)	0.024	0.186	0.852			
TG (mmol/L)	0.019	0.162	0.871			
ApoA1 (g/L)	0.159	0.321	0.748			
ApoB (g/L)	− 0.168	0.391	0.696			
Lp (a) (g/L)	0.001	0.087	0.931			
Creatinine (mmol/L)	− 0.003	0.520	0.603			
Uric acid (mmol/L)	0.001	0.854	0.394			
Fasting glucose (mmol/L)	0.185	1.693	0.091			
*Season classification*						
Spring VS Summer	0.320	0.738	0.461	0.262	0.614	0.540
Autumn VS Summer	0.022	0.056	0.955	0.046	0.119	0.905
Winter VS Summer	1.133	2.770	0.006	1.176	2.917	0.004

### Linear regression analysis of 24-h SBP coefficient of variation

The demographic and clinical characteristics and laboratory data of patients based on 24-h SBP coefficient of variation are presented in Table 7. Those with 24-h SBP coefficient of variation were mostly older, had differences in educational qualifications and triglycerides, and were admitted in winter (all *P* < 0.05; Table 7). Multivariate linear regression analysis showed that educational qualifications and admission in winter were independently associated with 24-h standard deviation of SBP (*P* < 0.05; Table 7).

**Table 7:** Univariate and multivariate linear regression models for 24-h SBP coefficient of variation

	Univariate			Multivariate		
	*β*	*t*	*P*	*β*	*t*	*P*
Female *n* (%)	0.001	0.227	0.821			
Age (years)	0.001	2.266	0.024	0.001	1.064	0.288
Diabetes mellitus	− 0.001	0.253	0.800			
Current smoker *n* (%)	− 0.003	0.866	0.387			
CAD *n* (%)	0.007	1.538	0.125			
PCI *n* (%)	− 0.003	0.380	0.704			
Previous myocardial infarction *n* (%)	− 0.012	1.360	0.175			
Family history of hypertension *n* (%)	− 0.010	1.479	0.140			
Educational Qualifications *n* (%)	− 0.003	3.074	0.002	− 0.003	2.368	0.018
BMI (kg/m^2^)	− 0.001	0.087	0.931			
hs-CRP (mg/L)	0.002	0.693	0.489			
HDL-C (mmol/L)	0.008	1.654	0.099			
LDL-C (mmol/L)	0.001	0.116	0.908			
TC (mmol/L)	0.001	0.015	0.988			
TG (mmol/L)	− 0.003	2.180	0.030	− 0.002	1.612	0.108
ApoA1 (g/L)	0.009	1.752	0.081			
ApoB (g/L)	− 0.004	0.955	0.340			
Lp (a) (g/L)	0.001	0.026	0.979			
Creatinine (mmol/L)	− 0.001	0.874	0.382			
Uric acid (mmol/L)	− 0.001	1.548	0.122			
Fasting glucose (mmol/L)	− 0.001	0.388	0.698			
*Season classification*						
Spring VS Summer	0.006	1.477	0.140	0.006	1.478	0.140
Autumn VS Summer	0.004	0.994	0.321	0.004	1.020	0.308
Winter VS Summer	0.014	3.478	0.001	0.015	3.670	< 0.001

### Linear regression analysis of 24-h DBP coefficient of variation

The demographic and clinical characteristics and laboratory data of patients based on 24-h DBP coefficient of variation are shown in Table 8. Those with 24-h DBP coefficient of variation were mostly older, had differences in diabetes mellitus, family history of hypertension, and higher educational qualifications, and were admitted in winter (all *P* < 0.05; Table 8). Multivariate linear regression analysis showed that age and admission in winter were independently associated with 24-h standard deviation of DBP (*P* < 0.05; Table 8).

**Table 8:** Univariate and multivariate linear regression models for 24-h DBP coefficient of variation

	Univariate			Multivariate		
	*β*	*t*	*P*	*β*	*t*	*P*
Female *n* (%)	− 0.002	0.596	0.551			
Age (years)	0.001	3.429	0.001	0.001	2.494	0.013
Diabetes mellitus	0.011	1.977	0.049	0.009	1.630	0.104
Current smoker *n* (%)	0.003	0.599	0.549			
CAD n (%)	0.010	1.610	0.108			
PCI *n* (%)	0.007	0.579	0.563			
Previous myocardial infarction *n* (%)	− 0.003	0.211	0.833			
Family history of hypertension *n* (%)	− 0.022	2.271	0.024	− 0.015	1.620	0.106
Educational Qualifications *n* (%)	− 0.004	2.657	0.008	− 0.002	1.349	0.178
BMI (kg/m^2^)	0.001	1.383	0.167			
hs-CRP (mg/L)	0.004	1.243	0.215			
HDL-C (mmol/L)	0.008	1.307	0.192			
LDL-C (mmol/L)	− 0.001	0.111	0.912			
TC (mmol/L)	− 0.001	0.518	0.604			
TG (mmol/L)	− 0.002	1.044	0.297			
ApoA1 (g/L)	0.006	0.934	0.351			
ApoB (g/L)	− 0.007	1.153	0.250			
Lp (a) (g/L)	0.001	0.297	0.767			
Creatinine (mmol/L)	− 0.001	0.642	0.521			
Uric acid (mmol/L)	0.001	0.909	0.364			
Fasting glucose (mmol/L)	0.002	1.425	0.155			
*Season classification*						
Spring VS Summer	0.008	1.244	0.214	0.006	1.064	0.288
Autumn VS Summer	0.003	0.611	0.541	0.003	0.590	0.555
Winter VS Summer	0.015	2.646	0.008	0.016	2.849	0.005

## Discussion

In this retrospective observational cohort study of patients with a first diagnosis of essential hypertension, we found that SBP, DBP, hs-CRP, SBP drop rate, DBP drop rate, 24-h standard deviation of SBP, 24-h standard deviation of DBP, 24-h SBP coefficient of variation, and 24-h DBP coefficient of variation were associated with new-onset essential hypertension in patients admitted in winter. It is noteworthy that those in the winter group had the highest 24-h standard deviation of SBP, 24-h standard deviation of DBP, 24-h SBP coefficient of variation, and 24-h DBP coefficient of variation.

### Underlying mechanisms of seasonal variations affecting new-onset essential hypertension

The significant effects of seasonal changes on cardiovascular diseases, including hypertension, have been well documented. In particular, BP has been reported to be higher in winter [17–19]. With seasonal shifts and corresponding temperature changes, there is a concomitant shift in the seasonal peaks of BP [20]. Epidemiological studies revealed that an inverse correlation between BP and temperature is likely the result of more physiological thermoregulation: vasoconstriction and increased peripheral resistance in cold environments, and vasodilatation and decreased peripheral vascular resistance in warm environments [21]. Consistent with the above findings, we found that SBP and DBP were highest in winter. Our findings are also consistent with previous findings showing that the circadian rhythm of BP fluctuation is affected by seasonal changes and temperatures, with a larger nocturnal BP decline and a more common dipping pattern in winter compared to summer [22]. We speculated that seasonal variation-linked BP changes are related to sympathetic nervous system activity, which is also affected by large seasonal changes in temperature [23]. In response to chronic cold exposure, circulating norepinephrine levels increase and are independently and negatively correlated with outdoor temperatures [23]. Our findings are inconsistent with one previous study showing that LF, as an index of sympathetic system activity, and HF, as an indicator of parasympathetic activity, were higher in summer and winter than in spring and autumn [15]. In the present study, we did not observe an association between 24-h average SBP and DBP and seasonal variations. It is likely that 24-h average BP was affected by stable ambient temperature modulated by an internal air conditioning system or floor heating system indoors. Notably, the winter group had the highest clinic BP among these four groups. This may be due to the fact that white coat effect leading to increased sympathetic nervous activity [24]. Moreover, in outpatient clinic setting, cold-induced peripheral vasoconstriction leading to a rise in peripheral vascular resistance may account for the increase in clinical BP with cold exposure. More research is needed to understand the sympathetic nervous activity that is affected by seasonal variations.

Hypertension is a multi-factorial chronic inflammatory disease, and a tight link between atherosclerosis and inflammation has emerged [25, 26]. The present study indicated that markers of inflammation were highest in winter. We found that changes in hs-CRP levels also varied seasonally and may be a potential mechanism underlying seasonal-related cases of new-onset essential hypertension. The inflammatory response is mediated to a large extent by oxidative stress and reactive oxygen species, which causes structural and functional changes in blood vessels and endothelial dysfunction, contributing to increased BP [27].

### Season and ambulatory BP

Seasonal BP variations are a global phenomenon that affect both sexes, all age groups, normotensive individuals, and untreated and treated hypertensive patients [3]. A previous cross-sectional study of 24 populations from 15 countries in Europe, Asia, and Australia revealed seasonal variations in SBP of 2.9 and 3.4 mmHg in the Northern and Southern Hemispheres, with the lowest points occurring in June and January, respectively [28]. In the present study, BP changed concomitantly with seasonal changes, but 24-h average SBP and DBP were not associated with seasonal variations. In contrast, other studies have revealed a stronger correlation between indoor temperatures and dynamic BP fluctuation compared to associations between outdoor temperatures and BP fluctuation [29]. The inconsistency between these studies is probably due to different criteria used for patient enrollment. It is also likely attributed to the fact that previous studies did not address the association between seasonal variations and new-onset essential hypertension as we did in this study. Importantly, the present study confirmed an association between BP variability and seasonal variations in new-onset essential hypertension. It is now widely accepted that BP variability, as a predictor of poor outcomes for stable coronary heart disease [30], is also a cardiovascular risk factor [31]. In this study, we found that standard deviation of SBP and DBP, 24-h SBP coefficient of variation, and 24-h DBP coefficient of variation were higher in winter than in summer, even after adjusting for comorbidities, which may explain why the incidence of cardiovascular events is affected in winter. Nevertheless, since we did not include weighted measures of BP variability, the “winter” associated differences in variability could be due to higher nighttime dipping during winter. Thus, a large difference between day and nighttime measurements would result in high standard deviation of SBP and DBP, 24-h SBP coefficient of variation, and 24-h DBP coefficient of variation over 24 h. On the basis of seasonal-linked BP fluctuations, ambulatory BP measurements can increase the comprehensiveness and accuracy of assessing BP changes during seasonal variations, which is conducive to making optimal treatment decisions. It should be noted that seasonal variations could be easily overlooked, leading to ineffective management of hypertension and potentially disastrous consequences. Therefore, the risk stratifications for new-onset essential hypertension patients require further optimization, particularly for identifying prognostic factors of unfavorable diseases.

### Study limitations

First, our work was a retrospective observational study with a relatively small sample size, and thus there could be selection bias in our study. Second, our results might have been influenced by other environmental factors including low atmospheric air pressure, high wind velocity, shorter sunshine duration, and air pollution. Although we chose to focus on environmental temperature in the present study, we cannot rule out the potential effects of other factors on the association between environmental temperature and BP fluctuations observed in this study. Third, all patients were divided into four groups based on the date of examination. Therefore, we did not account for individual differences in the current study. However, age, sex, and cardiovascular risk factors were not significantly different between these groups. Five, 24-h-weighted indices of BP variability were not analyzed [32], because of the retrospective nature of this study, the 24-h ambulatory BP monitoring was not digitally recorded. Therefore, we were unable to investigate all parameters in detail. Six, the 24-h ambulatory BP monitoring device used in this study has not been clinically validated. Seven, 24-h ambulatory ECG and BP monitoring device was different from the traditional method for 24-h ambulatory BP monitoring. Finally, in the present study, we did not perform multiple examinations. Hence, further studies with multiple testing are needed to corroborate our findings from this study.

## Conclusions

Winter was independently associated with the highest 24-h standard deviations of SBP, 24-h standard deviations of DBP, 24-h SBP coefficient of variation, and 24-h DBP coefficient of variation. It is important to integrate seasonal variations into clinical practice to make appropriate prevention and management decisions of new-onset essential hypertension.

## Data Availability

We collected the demographic data, clinical characteristics, risk factors, blood samples, biochemical data, data of 24-h ambulatory ECG and BP measurement in the People's Hospital of Xuancheng City between January 2019 and December 2019. The data that support the fndings of this study are available from the People's Hospital of Xuancheng City, but restrictions apply to the availability of these data, which were used under license for the current study, and so are not publicly available. The datasets used and/or analyzed during this study are available from the corresponding author on reasonable request. Requests to access these datasets should be directed to Jun Wang, 1,057,958,292@qq.com.

## Abbreviations

BMI: Body mass index
SBP: Systolic blood pressure
DBP: Diastolic blood pressure
TC: Total cholesterol
TG: Triglyceride
HDL-c: High-density lipoprotein cholesterol
LDL-c: Low-density lipoprotein-cholesterol
Apo-AI: Apolipoprotein A1
Apo-B: Apolipoprotein B
Lp(a): Lipoprotein (a)
CAD: Coronary artery disease
hs-CRP: High-sensitivity C-reactive protein
PCI: Percutaneous transluminal coronary intervention
SDNN: Standard deviation of normal R-R intervals
SDANN: Standard deviation average of NN intervals
rMSSD: Root mean square successive difference of normal R-R intervals
PNN50: Percent of the number of times that the difference between adjacent normal RR intervals > 50 ms in the total number of NN intervals
HF: High-frequency power
LF: Low-frequency power
